# Research on the Aging-Friendly Kitchen Based on Space Syntax Theory

**DOI:** 10.3390/ijerph19095393

**Published:** 2022-04-28

**Authors:** Ying Wang, Di Lin, Ze Huang

**Affiliations:** 1 Universal Design Institute, Zhejiang Sci-Tech University, Hangzhou 310018, China; linz10050317@163.com (D.L.); 6709437@163.com (Z.H.); 2 Silk and Fashion Culture Research Center of Zhejiang Province, Zhejiang Sci-Tech University, Hangzhou 310018, China

**Keywords:** space syntax, older adults, kitchen space, proper aging

## Abstract

As a result of aging populations globally, a growing number of older adults prefer to age in place; therefore, it is essential to study the spatial adaptability of the house. This study aimed to explore the relationship between the efficiency of daily activities and the spatial layout of home kitchens in the elderly population, and to assess the moderating role of cognitive function. Twenty-one elderly participants completed the experiment, including non-cognitively impaired (*n* = 12) and cognitively impaired groups (*n* = 9). Their home kitchen space was partitioned in plan and elevation based on space syntax theory. They were required to complete three tasks (i.e., an easy task, a medium task, and a difficult task) in their respective kitchens. The relationship between the efficiency of different tasks’ completion and the corresponding kitchen space attributes (integration, mean depth) was examined. The results showed a significant association between the home kitchen space layout of homebound older adults and their kitchen activity efficiency. In addition, a positive moderating effect of cognitive ability was found in the association between moderate and difficult tasks (*p* < 0.05), and its effect appeared to be stronger under challenging tasks (*p* < 0.01). The results of this study may help provide a basis for future design and optimization of aging-friendly residential spaces.

## 1. Introduction

As the problem of aging increases, a growing number of older adults prefer to stay in the community and live at home for a more extended period, compared to choosing a care facility in which to age [1]. This phenomenon is known as “aging in place” and is gradually becoming a global trend [2]. However, due to aging, cognitive and motor coordination deterioration prevents older adults from safely performing some daily activities at home [3]. Therefore, ensuring the flexibility and accessibility of residential environments for seniors adds to their sense of security in life and is also helping them to improve their ability to age in place [4].

Improvements to the residential environment for the elderly, especially modifications to the original structure and interior space design of their homes, are critical factors in promoting aging in place [5]. However, previous studies have found that many older adults still have many deficiencies in their family home space design, especially in active areas of daily life, such as bathrooms, kitchens, and dining rooms [6]. To address these issues, many researchers have studied the physical indoor environment of the elderly through fieldwork and qualitative interviews. However, less research has been conducted on the internal spatial structure of the homes of the elderly through quantitative means [7,8,9,10].

To date, space syntax theory has provided a new perspective for the quantitative study of space, making the research results more scientific and systematic through the presentation of graphics and data [11,12]. Most of the research in the exploration of areas related to the activities of older people has focused on public care and social environments. For example, Koohsari et al. [13] elucidated the association between street layout and cognitive function in Japanese older adults by combining objective physical activity assessments with the spatial attributes of streets in 277 Japanese older adults. Cao and Dewancker [14] used an empirical-based spatial zoning approach to analyze the design principles for the spatial layout of nursing homes, and Rodiek and Fried [15] studied the preferences of elderly residents for specific environmental characteristics of nursing homes. Mariana et al. [16], looking at the pattern of residential life forms in public spaces for the elderly, found that lobbies and receptions play an essential role in activity spaces for the elderly. Heo and Lee [17] studied nursing facility wards’ accessibility and identification by deriving their circulation structure. In contrast, only one study was previously identified that included a survey of the interior of a family home space for people with dementia, using convex space to analyze the relationship between overall home layout and older adults’ ability to perform daily living activities [18]. However, there are no empirical studies examining the association of single functional spaces with the behavioral activities of older adults, although the behavior in this part of life cannot be ignored.

The kitchen is one of the main activity centers of family life, and it is an essential part of the daily activities of the elderly [19,20]. Studies have shown that sustainable kitchen design is beneficial to the lives of older adults [21]. In addition, a more inclusive kitchen layout can make life easier for older adults [22]. However, the kitchen as a functional space, the existing standardized kitchen space layout, and backward forms of interaction in some households currently do not fully meet the characteristics of this group, adding to their physical activity burden [23]. Therefore, for most older adults to operate more comfortably, safely, and healthily in the home kitchen, it is necessary to analyze the layout of the kitchen space in conjunction with the behavior of aging users to provide support for aging in place [24].

The purpose of this study was to explore the association between the efficiency of the daily life of older adults at home and the spatial layout of their home kitchens, and to conduct an empirical study of the home kitchens of selected elderly users based on space syntax theory. The results of this study may provide support for the development of effective aging-in-place strategies and have implications for future research on more extensive aging-friendly spaces.

## 2. Methods

### 2.1. Participants

All participants involved in the study were Chinese older adults who lived in Hangzhou, Zhejiang Province, and all housing comprised residential apartments. They were recruited via poster advertisements at a hospital and senior activity center in their location from January to February 2021. The study inclusion criteria were as follows: (a) over 60 years old; (b) participant lives independently or with their spouse; (c) participants had a kitchen at home and must have experience using a kitchen; (d) participants did not suffer from any mobility impairment and were able to move freely without assistive devices such as wheelchairs or crutches; and (e) participants did not suffer from any sensory impairment and were able to communicate or interact with the researcher. Twenty-one participants met the inclusion criteria and agreed to participate. In addition, to better investigate the moderating effect of the cognitive abilities of older adults on the efficiency of their kitchen activities, each participant’s cognitive function was assessed using the Mini-Mental State Examination (MMSE) [25,26]. Ultimately, nine participants with scores ≤23 were included in the cognitively impaired group, whereas the remaining participants were included in the non-cognitively impaired group, taking into account the participants’ education and culture.

Regarding this experiment, the Institutional Review Board of the Zhejiang Sci-Tech University approved all procedures involving the participants. Before all experiments, to allow the subjects to participate in the experiment in the most natural state possible, the researchers informed the subjects of the safety of this experiment by showing the relevant documents and papers of the experimenters. The participants signed the informed consent form and the experimenters affirmed that all data involved in the study would be used only for the subsequent statistical analysis. All persons who declared their participation in the study signed the informed and voluntary consent form.

### 2.2. Space Syntax Theory

Space syntax theory was first introduced by Professor Bill Hillier and his colleagues at the University College London in the 1970s [27]. It describes the relationship between architecture and urban spatial patterns. Space syntax theory explains the accessibility and connectivity of spaces within a layout. In a domestic environment, space syntax measures how a small space is connected to the whole spatial system (integration) and the degree to which the entire system is divided into different spaces (depth). One of the main principles is to set the area in a closed space where the user spends most of his time as the starting node and use that node to complete subsequent metrics.

In this experiment, we obtained different kitchen space dimensions through field research measurements, and used AutoCAD (AutoCAD 2019, Autodesk, Inc., New York, NY, USA) to draw the dimensions of the kitchen spaces of the elderly study subjects. These dimensions were converted to dxf format, and space syntax analysis was finally conducted using DepthmapX (DepthmapX 0.8.0, UCL, London, UK) to produce space syntax measures of integration and mean depth [28]. Currently, DepthmapX can be used to conduct convex map analysis, visibility map analysis, and axial map analysis, thus providing quantitative and linear analysis of spatial fabrics. However, the convex map analysis method is more favorable for this study because of the small kitchen space inside the residence.

### 2.3. Experimental Design

**Figure 1:**
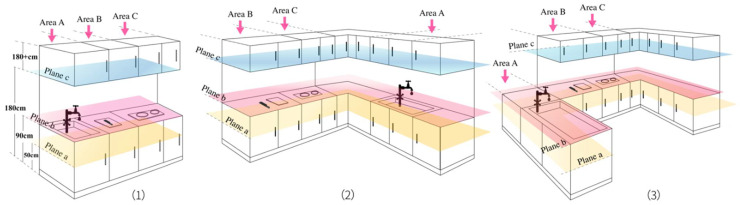
Different kitchen types’ zoning diagram. (1) I-shaped kitchen. (2) L-shaped kitchen. (3) U-shaped kitchen. Kitchen space façade partition is defined as (A) washing area, (B) preparation area, and (C) cooking area. Kitchen space plane partition height is defined as (a) yellow zone: bending posture storage space height of 50 cm; (b) red zone: normal standing posture, the ideal counter height of 90 cm; (c) blue zone: high cabinet storage space height of 180 cm or more.

The experimental scenario was the kitchen area in the subjects’ homes. Given the different subjects’ living space compositions and cognitive differences, some preparatory work needed to be completed before the experiment to deal with possible communication barriers and non-consistent task flows in the experimental process. In this study, to ensure the existence of objective and consistent task volumes for different participants, the layout of their kitchens was first categorized into the I-shape, U-shape, and L-shape, and then divided into a planar space and façade space according to the corresponding intrinsic layout. The planar space is independently partitioned according to the system function, whereas the façade space is partitioned according to the measured user’s body size with regard to the counter storage space (i.e., the convenient storage height of the human body in different postures is expressed as a percentage of the user’s height). In the bent posture, the user’s storage height ranges from 20% to 40%. In contrast, in the normal standing posture, the user’s tall counter storage height ranges from 110% or more of the user’s height, and the ideal counter height when naturally relaxed varies from 70 to 115 cm depending on the user’s height. This study is based on China’s GB-10000-88 “Chinese adult human body size” implemented in July 1988; the 50th percentile for men (i.e., 165 cm), combined with the 90th percentile heights for men and women, which were selected as a compromise, were used as an example for subsequent analysis (Figure 1).

**Figure 2:**
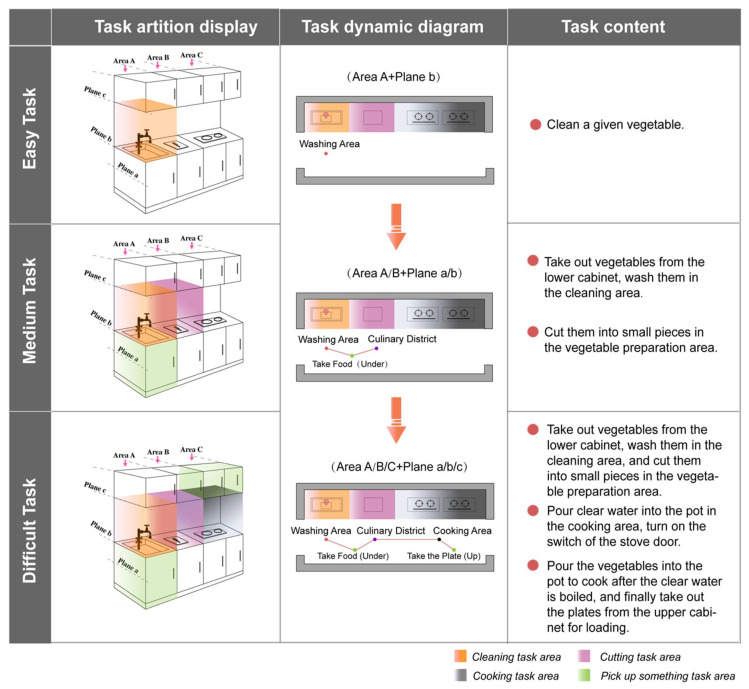
Examples of different tasks in the kitchen.

To better carry out the tasks, the kitchen space was divided into nine equivalent system areas according to the use functions through the plan and elevation zoning of the space. The tasks with equal difficulty in different kitchens were named according to the functional areas through which the participants completed the layered tasks, taking the I-shaped kitchen as an example (Figure 2).

### 2.4. Procedures

All experiments were conducted in the kitchen area of different participants’ homes. Upon the arrival of the researchers, all participants first completed a consent form and a short demographic survey. Then, each participant was required to perform a cognitive ability assessment using the Mini-Mental State Examination. During the experiment, participants were asked to perform three tasks of varying difficulty levels in sequence; participants took a break of one minute after completing each task and before moving on to the next task. Throughout the experiment, participants were allowed to ask of the experimenter, who accompanied the experiment. However, experiments they did not provide any assistance that might affect the experiment’s outcome (i.e., prompts, instructions, pausing the task, etc.). The entire experiment lasted approximately 15 min.

### 2.5. Data Analysis

The corresponding data were examined using SPSS 26.0 (IBM, Armonk, NY, USA). First, descriptive statistics were derived from the demographic data of the entire sample. Secondly, the Shapiro–Wilk test was utilized to test whether the objective variable data were normally distributed. Then, one-way ANOVA was used to analyze whether there was a significant difference between the task completion times to test whether the task difficulty settings were successful. The values of different task completion time variables were compared between the non-cognitively impaired and cognitively impaired groups using the independent samples *t*-test. Pearson correlation analysis was used to analyze the correlation between the different task completion times and the corresponding kitchen integration and mean depth. Specifically, the spatial attributes of the kitchen were characterized using the convex map analysis from space syntax theory. Finally, stepwise multiple regression analyses were performed to check whether there was an effect on user cognitive ability of kitchen space layout and task completion time. The significance level was set at *p* < 0.05 for all statistical tests.

## 3. Results

### 3.1. Sociodemographic Characteristics

Excluding the sample that were not available for subsequent analysis due to invalid experiments or missing data, the characteristics of the subjects are shown in Table 1. The mean age of the participants was 71.7 years; ten were male, and eleven were female. A total of nine subjects (42.9%) had mild or higher cognitive impairment (MMSE ≤ 23) based on the test for cognitive ability. Slightly less than one-third (28.6%) of the total number of subjects had received a higher level of education, and 42.9% of the subjects indicated the presence of two or more chronic conditions in their self-reported physical status.

### 3.2. Analysis of User Kitchen Space

**Figure 3:**
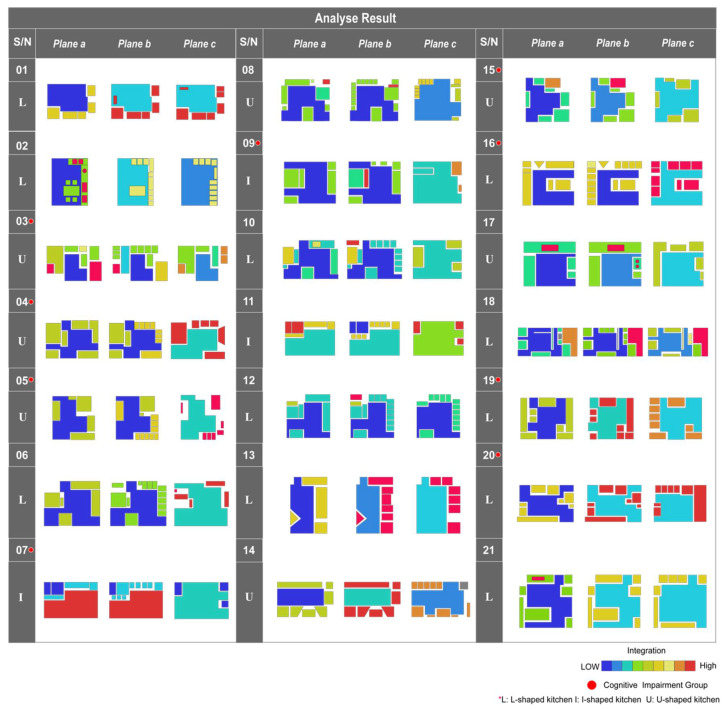
Kitchen sample space layout analysis.

According to the planar partition of the kitchen space, the kitchen space of the twenty-one participants’ homes was dimensionally extracted [29]. The kitchen plane corresponding to planes a, b, and c was depicted using AutoCAD and then imported into DepthmapX for convex map analysis. The warmer the color in the analysis results, the higher the corresponding spatial parameter value; conversely, the cooler the color, the lower the parameter value [30]. Our analysis found that the spatial layout of all kitchen samples contained 11 L-shaped cases, 7 U-shaped cases, and 3 I-shaped cases (Figure 3).

Table 2 shows all kitchen samples’ spatial attributes, which are also taken from the mean for better measurement. The result of the integration degree indicates the degree of clustering or dispersion of space from other spaces. The higher the value, the higher the accessibility within the space. The depth means the minimum number of connections that a space needs to pass through to reach other spaces; the higher the depth value, the lower the integration of the space, and, conversely, the lower the depth value, the higher the integration. According to the sample results, sample (02) had the highest overall kitchen depth of 3.5, followed by sample (12) with 3.06 and sample (19) with 2.99; all had L-shaped kitchen layouts. Samples with the highest integration were samples (04) and (05), both of which had U-shaped kitchen layouts with values of 1.76 and 1.69, respectively.

### 3.3. Analysis of the Correlation

**Figure 4:**
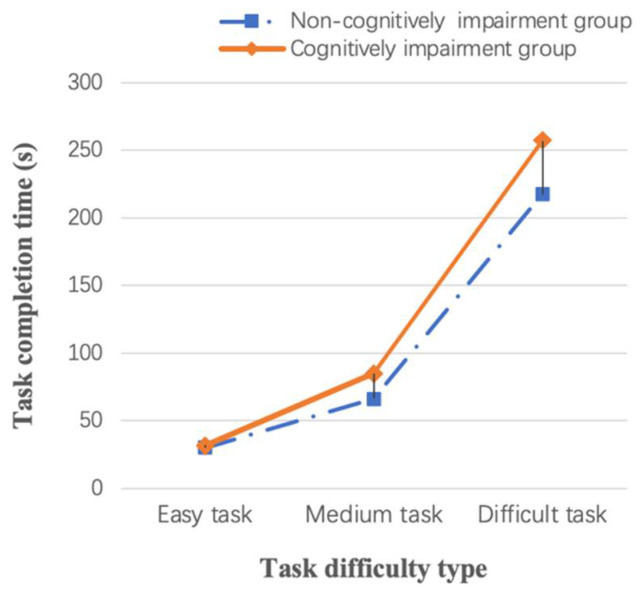
Mean value graph of task completion time for different groups.

Table 3 shows the results of the one-way ANOVA for the three task completion times. We found significant differences in the variable of completion time between tasks (F = 897.761, *p* < 0.05), which indicates that the task difficulty was successfully set in the pre-study period and can be used for subsequent predictive analyses. It is clear that, in the difficult task, older adults in the cognitively impaired group took longer to engage in the kitchen activity compared to the easy and medium tasks, which may be related to their weakened perceptual and comprehension abilities (Figure 4).

Table 4 shows the results of Pearson correlation analysis between spatial attributes and task completion time. T3 was found to be significantly negatively correlated with the mean depth of the kitchen space (r = −0.546, *p* < 0.05) and cognitive ability (r = −0.869, *p* < 0.01), and significantly positively correlated with integration (r = 0.825, *p* < 0.01). In addition, T2 was also positively correlated with integration (r = 0.563, *p* < 0.01) and negatively correlated with cognitive ability (r = −0.589, *p* < 0.01), but not significantly correlated with mean depth (r = −0.656, *p* > 0.05). T1 was not statistically significant in terms of mean depth and integration or cognitive ability.

Although there was no significant moderating effect of cognitive ability on older adults in the completion of simple tasks, in completing moderate and difficult tasks, cognitive ability played a significant positive role. Furthermore, cognitive ability was also highly correlated with integration and mean depth, suggesting that the layout of different home kitchen spaces affects older adults’ cognitive behavior to various degrees. This may also be related to the fact that the use of kitchen spaces with tighter functional areas by older adults with mental impairment may have a more positive impact on their residential life.

### 3.4. Differences between the Non-Cognitively Impaired and Cognitively Impaired Groups

To verify the different performances of the older non-cognitively impaired and older cognitively impaired groups in the same kitchen task, Table 5 shows the results of independent sample *t*-tests for both groups. The analysis showed no significant difference in the reaction time between the cognitively impaired and non-cognitively impaired groups in completing the simple task with a uniform number of tasks (t = −0.785, *p* > 0.05). However, the reaction times in the cognitively impaired group were statistically significantly higher in the completion of the medium task (t = −4.027, *p* < 0.05) and the difficult task (t = −5.594, *p* < 0.01) than in the non-cognitively impaired group, and this difference was more pronounced in the completion of the difficult task. Furthermore, correlating the kitchen sample analysis plots above reveals that some of the participants in the cognitively impaired group performed the task in the L-shaped layout kitchen. This may imply that the higher spatial depth of the L-shaped kitchen, i.e., the relatively scattered functional area layout, may further increase their cognitive load when completing complex tasks (Figure 3).

### 3.5. Multivariate Regression Analysis

Table 6 shows a stepwise multiple regression analysis using T2 data to verify the specific role of cognitive ability and integration. The dependent variable was T2 and the independent variable in model 1 was integration; this model explained 31.7% of the overall variance in T2 (F = 8.817, *p* < 0.01). The independent variable cognitive ability was retained in model 2, and it was found that it still influenced T2 (*p* < 0.05), and the model explained 35.5% of the variance. Thus, when older adults complete moderately difficult tasks, they are influenced by the specific kitchen space layout and cognitive ability.

Table 7 shows the results of a stepwise multiple regression analysis of the T3 data, which was the dependent variable. The independent variable in model 1 was mean depth, which explained 29.8% of the overall variance, and it can be seen that mean depth influenced T3 (F = 8.078, *p* < 0.05). In addition, two independent variables, integration and cognitive ability, were gradually added to models 2 and 3, which explained 69.3% and 78% of the variance, respectively. It was found that the inclusion of integration (F = 20.315, *p* < 0.01) and cognitive ability (F = 20.118, *p* < 0.01) continued to affect T3 and improved the model’s fit.

The contrast revealed that the moderating effect of cognitive ability showed a higher degree of significance during difficult tasks than moderate tasks. Older users may need to invoke more active environmental perception and spatial understanding to disassemble or analyze the task when completing relatively difficult tasks, thus reinforcing the positive effect of cognitive ability across variables. Therefore, we can affirm that, although older users’ cognitive ability results in an impact on their behavioral level, when the regional accessibility of the space in the home kitchen layout is higher, i.e., there are more explicit or more cued paths for users to access different functional areas, it means that they have a more active ability to efficiently carry out daily living tasks.

## 4. Discussion

This study aimed to examine the association between kitchen space layout with different characteristics and the activity efficiency of kitchen tasks for elderly users. Specifically, the associations between integration and mean depth in different kitchen spaces and activity efficiency of elderly users were investigated, and the mediating role of cognitive abilities in this was explored.

Studies have shown that the layout characteristics of the home kitchen space affect the activities of daily living of older adults to some extent [22,31,32,33]. In addition, older adults with cognitive impairment are more likely to be affected by low activity efficiency due to the layout of the kitchen space. The data suggest that older adults are more likely to be affected by the spatial arrangement when they face challenging tasks in kitchen tasks, than when completing moderate and straightforward tasks. This implies that older adults need to invoke more active spatial perception abilities when completing complex daily activities to effectively perform the disassembly and analysis of that activity. Notably, this study found an association between the integration and mean depth of the kitchen space and the cognitive abilities of older adults, which supports several previous studies on the positive effects of good spatial layout on the daily living activities and cognitive abilities of older adults [34,35,36,37]. Therefore, when older adults with cognitive impairments use a highly integrated kitchen space layout, it may significantly reduce the complexity of individual activities in which older adults are involved. Overall, better accessibility and convenience in the layout of functional areas of the kitchen space may become essential considerations for the aging-in-place community in future space modifications.

This study found that older adults’ cognitive abilities significantly affect their activity efficiency when completing medium and difficult kitchen tasks. More importantly, when older users are in kitchen spaces that are less integrated, differences in cognitive ability levels can further affect their daily activity efficiency. Previous research has shown that older adults with cognitive impairments can be more vulnerable to the restrictive nature of built housing interiors, which can lead to marginalized living [38]. In addition, the layout of the housing space will also affect the physical and mental health of the elderly [39,40,41]. The design of the space with specific functional guidance can make them become more independent and help improve their sense of well-being [42]. Therefore, a clear division of functional areas in the kitchen is beneficial to alleviating the spatial cognitive burden of the elderly.

Our findings are consistent with some previous studies, but there are some differences [13,18,43]. A kitchen layout with more concentrated functional areas would gain more spatial accessibility [44]. It could better assist older users in memory and exploration, but this needs to be based on their familiarity with the environment [45]. In addition, the layout of the kitchen space affects the spatial cognitive ability of the users. Therefore, both will require more integration adaptation time to initiate activities in a newly established and maintained kitchen space for older adults. This is consistent with the findings of a previous study that showed older adults’ ability to interact in life is more likely to be influenced by environmental familiarity [46,47]. However, previous studies did not address the effects of spatial structure within the home on older adults’ behavioral competence.

This study found that partitioning the kitchen space of aging users based on space syntax can reveal a causal relationship between the layout and the efficiency of their activities. Specifically, when a kitchen space has a high value of integration, the paths to reach the different functional areas within the kitchen are closer. As a result, the efficiency of kitchen activities will also increase for older adults. The more decentralized the spatial kitchen system, the more complex the different functional areas involved in the subjects’ task completion, thus increasing the completion time. As previously mentioned, the increase in depth in the spatial system will increase the burden of spatial representation for older users, but will also impact their ability to live independently [48,49,50], and this is more evident in the L-shaped kitchen layout. Therefore, it may provide designers with new design ideas if the behavioral skills, environmental familiarity [51], and overall spatial layout of elderly users are linked to kitchen space improvement. We suggest that appropriate indicative signs can be considered in the kitchen space, such as improving the color or layout of lights, walls, switches, floors, etc., to better assist aging users in switching between different functional areas and reduce their operational risks [52]. In addition, assessing age users’ behavioral habits and cognitive abilities should be prioritized before designing to address their latent needs and enhance the environmental resilience and aging-in-place of older adults [53]. Furthermore, this may be an opportunity to provide relevant support for future government and related agencies to propose more aging-appropriate retrofitting decisions [54].

## 5. Conclusions

This study aimed to explore the association between the spatial layout of the home kitchen of elderly people and its activity efficiency. The study results showed that the level of integration and mean depth in kitchen space layout were associated with the activity efficiency of elderly users. In addition, the different cognitive levels of older adults moderated their activity efficiency in kitchen spaces, which was more pronounced when they performed difficult tasks. Based on these findings, we found that the methodology can also be applied to future studies to help support the analysis of elderly-oriented spaces.

From the aspect of promoting age-appropriate retrofitting of family residential spaces, research thinking based on space syntax theory presents a new perspective for analyzing the spatial layout of elderly households. This paper provides further references for designers in the future accessibility assessment of different interior spaces through a combination of theoretical and empirical data approaches, and provides information about the application of relevant design strategies. It is worth mentioning that this can better meet the residential needs of the elderly and adapt to their actual activities, thus providing for aging in place.

Future studies can explore the effects of a greater number of different types of domestic kitchen spaces on older adults, and extend the analysis to the other domestic areas, such as bathrooms, dining rooms, and bedrooms. In addition, since the kitchen spaces in this study were all from residential apartments in China, it is unclear whether these findings can be generalized to the home layouts of other ethnic and cultural groups, and more research is needed to test them. It is worth mentioning that the spatial syntactic model should be further integrated with the individual perceived home domains to take more account of the domestic factors of the homebound elderly (e.g., family attachment, family and friend care, memories, and values), and to work on building a model to help practitioners in the design or architectural planning industry to design and optimize home spaces and solutions for the elderly.

There were still some limitations to this study. First, there was a more prominent potential source of bias in the study: the greater likelihood of physical environmental changes in the subjects’ homes before the researchers’ spatial layout measurements. Our best guess is that all of the older participants accepted the researcher’s request regarding the needs related to environmental factors. Some of the subjects had undergone clutter removal or kitchen renovation before the researcher’s visit, which would have led to some changes in the corresponding space syntax assessment. Secondly, since the kitchen space of each subject household was different and there were inconsistencies in the kitchen triangle, in this study of the activity efficiency of the elderly, the researchers could only complete the planning of the total number of tasks by dividing the kitchen space into flat and elevation areas. Thus, there may still be some differences in the number of tasks, which are unavoidable. In addition, we recruited participants in the cognitive impairment group who were older and less educated than those in the non-cognitive impairment group, which may have resulted in bias in the results. Further research is therefore needed to understand the implications.

## Figures and Tables

**Table 1:** Characteristic information of experimental participants.

Variables		Non-Cognitive Impairment (*n* = 12)		Cognitive Impairment (*n* = 9)	
		*n*	%	*n*	%
Gender	Male	6	50.0	4	44.4
	Female	6	50.0	5	55.6
Age	M(SD)	69.2(2.31)		75.1(3.18)	
Education Attainment	Below middle school	8	66.7	7	77.8
	middle school or higher	4	33.3	2	22.2
Number of chronic diseases	≤1	6	50.0	6	66.7
	≥2	6	50.0	3	33.3

**Table 2:** Mean depth and integration properties of different kitchens.

Sample	Integration Value	Depth Value (M)	Sample	Integration Value	Depth Value (M)
01	1.37	2.69	12	1.04	3.06
02	0.99	3.50	13	1.35	2.71
03	1.60	2.61	14	1.31	2.68
04	1.76	2.58	15	1.52	2.65
05	1.69	2.50	16	1.49	2.91
06	1.38	2.53	17	1.31	2.79
07	1.67	2.54	18	1.44	2.83
08	1.49	2.98	19	1.12	2.99
09	1.64	2.58	20	1.52	2.77
10	1.35	2.70	21	1.59	2.70
11	1.39	2.61			

**Table 3:** One-way analysis of variance (ANOVA) for task completion time.

	SS	Df	MS	F	*p*-Value
Between groups	481,202.889	2	240,601.444	897.761	<0.01
Within group	16,080.095	60	268.002		
Total	0.299	62			

**Table 4:** Analysis of correlation between the different task completion times and spatial properties.

Variables	1	2	3	4	5	6
1. T1	—					
2. T2	−0.147	—				
3. T3	0.299	0.564 **	—			
4. Mean depth	−0.152	−0.656	−0.546 *	—		
5. Integration	0.239	0.563 **	0.825 **	0.651 **	—	
6. MMSE	−0.121	−0.589 **	−0.869 **	0.659 **	−0.886 **	—

* *p* < 0.05; ** *p* < 0.01; Abbreviations: T1 = easy task completion time; T2 = medium task completion time; T3 = difficult task completion time; MMSE = Mini-Mental State Examination.

**Table 5:** Differences in time to completion of different tasks between users in the non-cognitively impaired and cognitively impaired groups.

Variables	Non-Cognitive Impairment Group (*n* = 12)		Cognitive Impairment Group (*n* = 9)		t	*p*-Value
	Mean	SD	Mean	SD		
T1	30.00	6.03	32.00	5.41	−0.785	0.44
T2	66.42	11.02	85.00	9.64	−4.027	<0.05
T3	217.75	3.36	256.00	17.85	−5.594	<0.01

Abbreviations: T1 = easy task completion time; T2 = medium task completion time; T3 = difficult task completion time.

**Table 6:** A stepwise multiple regression to estimate the association between integration, cognitive ability, and T2.

Model	R	R^2^	Adjusted R^2^	F	*p*-Value
1	0.563	0.317	0.281	8.817	<0.01
2	0.596	0.355	0.284	4.959	<0.05

1. Predictors: (Constant), Integration.; 2. Predictors: (Constant), integration, cognitive ability (MMSE); Abbreviations: T2 = medium task completion time.

**Table 7:** A stepwise multiple regression to estimate the association between integration, mean depth, cognitive ability, and T3.

Model	R	R^2^	Adjusted R^2^	F	*p*-Value
1	0.546	0.298	0.261	8.078	<0.05
2	0.832	0.693	0.659	20.315	<0.01
3	0.883	0.780	0.741	20.118	<0.01

1. Predictors: (Constant), mean depth; 2. Predictors: (Constant), mean depth, integration; 3. Predictors: (Constant), mean depth, integration, cognitive ability (MMSE); Abbreviations: T3 = difficult task completion time.

## Data Availability

The data are not publicly available due to the data also forming part of an ongoing study.
